# Schwann Cell Activity in the Multiple Sclerosis Microenvironment

**DOI:** 10.3390/cells15171570

**Published:** 2026-08-29

**Authors:** Michael R. Shurin, Galina V. Shurin, Carolina Moreira Doyle, Anna E. Lokshin, Sarah E. Wheeler

**Affiliations:** 1 Departments of Pathology, University of Pittsburgh and University of Pittsburgh Medical Center, Pittsburgh, PA 15213, USA; shuringalina787@gmail.com (G.V.S.); wheelerse3@upmc.edu (S.E.W.); 2 Departments of Immunology, University of Pittsburgh and University of Pittsburgh Medical Center, Pittsburgh, PA 15213, USA; 3 Hillman Cancer Center, University of Pittsburgh and University of Pittsburgh Medical Center, Pittsburgh, PA 15213, USA; moreiradoylec@upmc.edu (C.M.D.); loksax@upmc.edu (A.E.L.); 4 Departments of Medicine, University of Pittsburgh and University of Pittsburgh Medical Center, Pittsburgh, PA 15213, USA

**Keywords:** Schwann cells, multiple sclerosis, intracellular signaling, transcription factors, chemotaxis

## Abstract

**Highlights:**

**What are the main findings?**

- Phenotypic, functional, and molecular analyses of human Schwann cells in the model multiple sclerosis microenvironment revealed that Schwann cell proliferation and motility were suppressed, while the expression of both pro-myelinating genes and negative regulators of myelination was up-regulated.
- The uncommon pattern of cellular signaling reprogramming, SC injury response, was associated with active phosphorylation of ERK and c-Jun and could be partially prevented by inhibitors of c-Jun and ERK1/2.

**What are the implications of the main findings?**

- These data not only demonstrate that Schwann cells undergo abnormal adaptive cellular reprogramming in the multiple sclerosis microenvironment but also suggest that they could be pharmacologically modified to enhance their potential for multiple sclerosis treatment.
- These findings lay the groundwork for developing new glia-focused treatments to fight axonal loss, pain, and neuroinflammation in multiple sclerosis.

**Abstract:**

Schwann cell (SC)-based therapy is currently being debated as an approach to promote functional recovery in patients with multiple sclerosis (MS) and other inflammatory demyelinating diseases of the central nervous system (CNS). The main limitation of SC transplantation in MS patients is the short-term functional activity of SCs in the CNS environment. The goal of this study was to determine phenotypic, functional, and signaling changes in human SCs treated with CSF samples from MS patients in vitro, and to characterize the molecular mechanisms underlying SC injury response in the model MS microenvironment. We demonstrated that SC proliferation and motility were suppressed, while the expression of both pro-myelinating genes and negative regulators of myelination was up-regulated in cells incubated with CSF from MS patients. This was associated with active phosphorylation of ERK and c-Jun, and inhibition of these signaling pathways prevented SC changes. The overall analysis of detected abnormalities and SC markers indicates that SCs do not exhibit either a ‘classic’ dedifferentiation-repair-like phenotype or a myelin-forming maturation phenotype when placed in MS-like conditions. They demonstrate an uncommon pattern of cellular signaling reprogramming, associated with decreased motility and potentially decreased myelination. We thus suggest that ERK- and JNK-modulated SCs should be further investigated as a potential cell source for CNS repair in MS.

## 1. Introduction

Schwann cells (SCs) are glial cells of the peripheral nervous system (PNS) that provide mechanical, metabolic, and functional support to neurons. In addition to their vital role in normal nerve physiology, SCs, thanks to their functional plasticity, coordinate repair mechanisms in various pathophysiological conditions and PNS diseases [1]. The response of SCs to neurotrauma, for instance, involves adaptive cellular reprogramming, leading to cell activation, denervation, dedifferentiation, proliferation, increased motility, and polarization toward a repair SC phenotype [2,3]. On the other side, different neuropathies, associated with traumatic, toxic, metabolic, infectious, inherited, and inflammatory conditions, can also involve morphological flexibility, epithelial–mesenchymal transition, stemness, and autophagic transformation of SCs, along with loss of myelin differentiation, myelin breakdown, ultrastructural abnormalities, altered motility and proliferation, and cell death [4,5,6].

The unique roles of SCs in regeneration, remyelination, and axon regrowth in the PNS offer opportunities to develop new therapeutic tools, including the transplantation of cultured or genetically modified SCs and the use of SC-derived factors, such as ECM proteins, growth factors, and exosomes [5]. Supplementing SCs at injured sites is considered a feasible therapeutic approach for severe peripheral nerve injuries [7]. However, SCs can also contribute to the CNS regenerative response. Although SCs do not naturally occur in the CNS, some data suggest that SCs from the PNS can be recruited to aid myelin repair after CNS injury [8,9,10,11]. The benefits of transplanting cultured SCs for spinal cord injury therapy have been extensively studied over the past few decades in animal models and clinical trials. It has been shown that administering SCs into the injured spinal cord reduces tissue loss, supports the survival of damaged neurons, promotes axonal regeneration, and speeds up myelination, along with functional recovery [12,13,14,15]. Additionally, the role of resident SCs in disease processes and the reparative effects of transplanted SCs in various non-traumatic CNS demyelinating diseases, such as multiple sclerosis, leukodystrophies, amyotrophic lateral sclerosis, and others, have been well documented [5].

Multiple sclerosis (MS), an autoimmune disease characterized by focal demyelinated lesions in the CNS, is a significant cause of neurological disability worldwide, with increasing prevalence in many countries [16]. Because physiological remyelination in MS has been demonstrated [17,18], therapeutic induction and stimulation of remyelination in MS lesions may offer a new, viable treatment approach [19]. Preclinical studies using experimental autoimmune encephalomyelitis (EAE) in rodents showed that transplanting SCs into demyelinated areas significantly enhances myelin repair and regeneration [20,21,22,23]. Additionally, reports of SC remyelination in the cerebrum, brainstem, and spinal cord of autopsied and biopsied MS cases suggest that SCs could contribute to CNS repair [24,25,26,27]. This evidence of gliogenesis in the lesioned CNS is clinically important because it indicates that remyelination can repair damaged CNS regions, offering a potential treatment strategy for MS. It also highlights the role of regeneration-promoting cells that could be used in therapy. SCs are among the most promising candidates for autologous transplantation to repair myelin [19,20,28].

Despite SCs’ indicated reparative capacity, they are poorly tolerated in the CNS after transplantation due to various factors within the hostile microenvironment of lesioned tissue [2,29]. For example, the motility, long-term survival, and remyelination of some injured CNS axons by grafted SCs may be impaired near the transplantation site [30,31]. Therefore, modifications of the exogenous SCs or the CNS environment are necessary to overcome the limitations of SC transplantation. No previous data have convincingly demonstrated potential abnormalities in SC function in MS, nor have they identified opportunities to prevent or correct these defects to improve SC-based therapies for MS [5]. As a result, it is essential to determine whether SCs are dysfunctional in MS and which signal transduction pathway is responsible. Additionally, it is important to explore whether targeting these signaling pathways can prevent SC alterations in the MS environment.

This study aimed to provide the first proof of principle that MS-associated factors in CSF or serum can regulate human SC function through specific signal transduction pathways. By analyzing the behavior and signaling of human SCs cultured with serum and CSF from patients with MS, we demonstrated changes in longevity and identified specific signaling pathways involved in SCs within the MS microenvironment. Identifying the signaling pathways that govern SC longevity and motility in the MS CNS is essential for developing effective treatment strategies to harness SCs for CNS repair and to slow or reverse disease progression. Our findings lay the groundwork for developing new glia-focused treatments to combat axonal loss, pain, and neuroinflammation in MS.

## 2. Materials and Methods

### 2.1. Schwann Cell Culture

Human SC line hTERT ipn02.3 2λ (CRL-3392) was obtained from ATCC (Manassas, VA, USA). These normal (NF1 wild-type, +/+) primary SCs were isolated from the sural nerve of a healthy female with no disease indications and immortalized by transduction with mCdk4 cDNA and *hTERT*. Cells were cultured in Dulbecco’s modified Eagle’s medium (DMEM, High Glucose, ATCC) supplemented with 10% FBS (GeminiBio LLC, Sacramento, CA, USA), 0.2 mM L-glutamine, 50 µg/mL penicillin, and 50 μg/mL streptomycin (ATCC). Cells were maintained at 37 °C in a humidified atmosphere containing 5% CO_2_. All experiments were conducted using verified mycoplasma-free cells. These SCs have been characterized earlier [32,33,34,35,36].

For SC harvesting in cultures, 0.25% Trypsin-EDTA was added to the flasks until cells detached; then an equal volume of DMEM medium containing 10% FCS was added to neutralize the enzyme’s effects. The cell suspension was centrifuged at 400× *g* for 5 min and resuspended in a smaller volume of medium. Cell number was determined using the hemocytometer and Trypan Blue staining (Sigma-Aldrich Inc., Burlington, MA, USA).

### 2.2. Serum and CSF Samples

This study was approved by the University of Pittsburgh IRB (STUDY23040014). The University of Pittsburgh has a Federal Wide Assurance approved through the Office for Human Research Protections (FWA00006790).

All CSF and serum specimens were remnant samples from clinical testing ordered as part of standard care. All CSF and paired serum samples were tested for the presence of CSF-unique oligoclonal bands (OCB) by Helena’s SPIFE Touch IgG Isoelectric Focusing according to the manufacturer’s protocol (Helena Laboratories, Beaumont, TX, USA). Concentrations of serum and CSF albumin and IgG were detected by turbidimetry on the Optilite Analyzer according to the manufacturer’s protocols (Binding Site Inc., San Diego, CA, USA). The IgG index, as a marker of intrathecal immunoglobulin synthesis, and the albumin ratio, as a marker of blood–brain barrier integrity, were calculated. The albumin ratio compares the concentration of albumin in the CSF to that in the serum. The IgG index evaluates the concentration of IgG antibodies in CSF relative to serum IgG levels, adjusted for albumin to account for blood–brain barrier permeability. All tests were performed at the College of American Pathologists-accredited, CLIA-certified UPMC Clinical Immunopathology Laboratory.

All samples were separated into two groups: Control (36 samples) and “MS” (35 samples). The following selection criteria were used. All Control CSF samples had an IgG Index lower than 0.7 (normal), a normal Albumin ratio (<15 y/o—<6; 16–60 y/o—<7; >60 y/o—<9), and no OCB. All “MS” samples had increased IgG Index, normal Albumin ratio, and >3 OCB.

The control group characteristics were: 30 females and 6 males, with a mean and median age of 33.8 and 34 for females (range: 12–80 years) and 35.5 and 33.5 for males (range: 14–58 years), respectively. The mean and median ages for the entire control group were 34 years. The “MS” group characteristics were: 25 females and 10 males, with mean and median ages of 39.7 and 40 for females (range: 20–79 years) and 46.3 and 46 for males (range: 12–80 years), respectively. The mean and median ages for the entire “MS” group were 41.6 and 41 years, respectively. The mean ± SD and median OCB numbers for females were 13.9 ± 5.0 and 13, respectively (range: 3–24). The mean ± SD and median OCB numbers for males were 12.8 ± 5.6 and 10.5, respectively (range: 7–25). The mean ± SD and median OCB numbers for the entire “MS” group were 13.6 ± 5.2 and 13, respectively. No statistically significant correlation between the number of OCB and age was detected (R = 0.13, *p* = 0.46).

Due to the limited volume of CSF samples available for SC cultures, many repetitive experiments used different CSF samples, but all from the same group. All sample types after OCB, albumin, and IgG testing were stored at −30 °C for 0.1–3 months before use in SC cultures or other assays.

### 2.3. Experimental Design

Cultured human SCs were co-incubated with CSF and serum samples obtained from patients with MS whose specimens were tested for OCB, IgG Index, and Albumin Ratio, as described above. Cells were incubated with “Control” (no OCB) and “MS” (OCB present) CSF and paired serum samples at 10% (*v*/*v*) in 25-cm^2^ flasks for RNA isolation and phenotyping, 6-well plates for Western blotting, 24-well plates for cell migration assay and for the detection of human neurotrophic factors, and 96-well plates for MTT assay. Control SCs were incubated with medium alone, washed, re-cultured, centrifuged, and analyzed in assays similar to those performed on experimentally treated SCs, as indicated in the individual methodological sections below.

### 2.4. Cell Proliferation/Activation Assay

The MTT assay, which is based on the conversion of MTT (3-[4,5-dimethylthiazol-2-yl]-2,5-diphenyltetrazolium bromide) into formazan crystals by living cells, and thus determines mitochondrial activity reflecting cell proliferation and activation, was used to characterize the longevity of SCs. To examine the proliferative activity of treated and control SCs, 5 × 10^3^ cells/well were seeded in 96-well plates and incubated for 48 h in culture medium. Following appropriate treatments, 1 mg/mL MTT reagent (Sigma-Aldrich) was added to each well for 4 h at 37 °C. The cells were then suspended in dimethyl sulfoxide for 3 h at 37 °C, and the optical density (OD) was determined on a Benchmark Microplate Reader (Bio-Rad, Hercules, CA, USA) at 570 nm. Quadruplicate data were used in all experiments.

### 2.5. Cell Migration/Chemotaxis Assay

SC motility was assessed using a QCM colorimetric transwell migration assay (Sigma-Aldrich) based on the Boyden chamber principle. Control and treated SCs, cultured in 25 cm^2^ flasks for 48 h, were harvested, centrifuged at 400× *g* for 5 min, and prepared as 0.25 × 10^6^ cells/mL. 500 µL of prewarmed DMEM with 10% FBS or control serum-free medium was added to the lower chamber of 24-well plates. A total volume of 250 µL of control and treated SC suspension was added to the insert (8 µm pore size). Cells were incubated for 24 h at 37 °C in a CO_2_ incubator. After incubation, the culture medium and cell suspension were removed from the top of the inserts, and the inserts were placed into clean wells containing 400 µL of Cell Stain for 20 min. Inserts were then rinsed with water, and non-invaded cells were gently removed with a cotton-tipped swab. Stained inserts were transferred to a clean well containing 300 µL of extraction buffer for 15 min. A dye mixture (100 µL, in duplicate) was transferred to a 96-well plate for colorimetric OD measurement at 560 nm on a Benchmark Microplate Reader (Bio-Rad). All functional experiments were repeated at least three times.

To check the involvement of ERK and c-Jun signaling pathways in the regulation of SC chemotaxis, cells were first pretreated for 2 h with 10 µM of ERK inhibitor UO126 (Cell Signaling Technology, Boston, MA, USA) or 10 µM JNK inhibitor SP600125 (Santa Cruz Biotechnology Inc., Dallas, TX, USA), followed by the addition of SCF samples or control medium. Inhibitor concentrations have been established in a series of pilot experiments.

### 2.6. Detection of Neurotrophic Factors

The ProcartaPlex Human Neurotrophic Factors Panel 4plex (Thermo Fisher Scientific Inc., Waltham, MA, USA) was used to assess the expression of BDNF, CNTF, GDNF, and NGF by control and treated SCs. SCs were seeded in 24-well plates at 0.25 × 10^6^ cells/mL and treated with 10 µM of an ERK or JNK inhibitor or with medium (control). Control medium or SCF samples (10% *v*/*v*) were added 2 h later. After 24 h of co-incubation, plates were centrifuged at 400× *g* for 10 min, and cell-free supernatants were collected. Concentrations of neurotrophic factors were measured in supernatants on the Luminex LabMAP system (Luminex Corporation, Austin, TX, USA) according to the manufacturer’s protocol. All samples were tested in duplicate, and all experiments were repeated twice.

### 2.7. RNA Isolation and Real-Time qPCR

SCs were seeded in 25 cm^2^ flasks at 0.25 × 10^6^ cells per 5 mL. Control medium or CSF and paired serum (10% *v*/*v*) were added to SC cultures for 48 h before cells were harvested. Total RNA was isolated by oligo-dT affinity (RNeasy, Qiagen, Germantown, MD, USA); cDNA was synthesized with Oligo (dT) and Moloney murine leukemia virus reverse transcriptase (Superscript III; Invitrogen, Carlsbad, CA, USA). Real-time PCR was performed on a MX3000P instrument (Stratagene, La Jolla, CA, USA) using SYBR Green to monitor PCR product accumulation. Reactions were performed in duplicate in 20 μL reaction volumes with 10 μL of pre-mixed dye, NTPs, buffer, and polymerase (SYBR Green Master Mix, Stratagene, La Jolla, CA, USA), to which 250 nM primers and 3 μL of first-strand cDNA were added. After 10 min at 95 °C, the mixture was amplified in cycles of 35 s at 95 °C, 30 s at 56 °C, and 1 min at 72 °C. Product abundance relative to controls was calculated assuming a linear log relationship (initial copies). Expression of the following transcription factors and other molecules in SC was assessed: Inhibitor of DNA binding protein 2 (ID2), early growth response 2 (EGR2), early growth response 1 (EGR1), SRY (sex-determining region Y)-box 2 (SOX2), SRY-box 10 (SOX10), POU domain, class 3, transcription factor 1 (Oct-6), T-box 2 (TBX2), c-Jun, nerve growth factor receptor (NGFR), glial fibrillary acidic protein (GFAP), neural cell adhesion molecule 1 (NCAM1), S100 calcium-binding protein B (S100B), myelin basic protein (MBP), and myelin associated glycoprotein (MAG). All qPCR experiments were repeated at least four times.

The following primers were used: GAPDH: Forward 5′-GGAGCGAGATCCCTCCAAAAT-3′; Reverse 3′-GGCTGTTGTCATACTTCTCATGG-5′ (Product size 197 bp). ID2: Forward 5′-GCTATACAACATGAACGACTGCT-3′; Reverse 3′-AATAGTGGGATGCGAGTCCAG-5′ (Product size 151 bp). EGR2: Forward 5′-TCTTCCCAATGATCCCAGACT-3′; Reverse 3′-TTACGGATTGTAGAGAGTGGAGT-5′ (Product size 154 bp). EGR1: Forward 5′-GGTCAGTGGCCTAGTGAGC-3′; Reverse 3′-GTGCCGCTGAGTAAATGGGA-5′ (Product size 149 bp). SOX2: Forward 5′-TACAGCATGTCCTACTCGCAG-3′; Reverse 3′-GAGGAAGAGGTAACCACAGGG-5′ (Product size 110 bp). SOX10: Forward 5′-CCTCACAGATCGCCTACACC-3′; Reverse 3′-CATATAGGAGAAGGCCGAGTAGA-5′ (Product size 161 bp). Oct-6: Forward 5′-AACGATAACATACCAAGCGTGT-3′; Reverse 3′-GGTCTGCCCGTTCAACATC-5′ (Product size 120 bp). TBX2: Forward 5′-GCTGACGATTGCCGCTATAAG-3′; Reverse 3′-GGCTGTCTGGGTGGATGTA-5′ (Product size 100 bp). NGFR: Forward 5′-CCTACGGCTACTACCAGGATG-3′; Reverse 3′-CACACGGTGTTCTGCTTGT-5′ (Product size 109 bp). GFAP: Forward 5′-GACCTGGCCACTGTGAGG-3′; Reverse 3′-GGCTTCATCTGCTTCCTGTC-5′ (Product size 209 bp). NCAM1: Forward 5′-GGCATTTACAAGTGTGTGGTTAC-3′; Reverse 3′-TTGGCGCATTCTTGAACATGA-5′ (Product size 100 bp). S100: Forward 5′-TGGCCCTCATCGACGTTTTC-3′; Reverse 3′-ATGTTCAAAGAACTCGTGGCA-3′ (Product size 248 bp). MBP: Forward 5′-GGCCGGACCCAAGATGAAAA-3′; Reverse 3′-CCCCAGCTAAATCTGCTCAGG-5′ (Product size 119 bp). MAG: Forward 5′-CCAAGTAGTCCACGAGAGCTT-3′; Reverse 3′-CAGGTCCCCACGGAAGTAGT-5′ (Product size 130 bp). JUN: Forward 5′-TCCAAGTGCCGAAAAAGGAAG-3′; Reverse 3′-CGAGTTCTGAGCTTTCAAGGT-5′ (Product size 78 bp).

### 2.8. Flow Cytometry

SCs were treated with control medium or CSF and paired serum for 48 h, harvested, and phenotyped by flow cytometry. To assess the expression of specific proteins, SCs were fixed and permeabilized using the BD Bioscience kit, followed by staining with anti-NGFR antibody conjugated with FITC (5 µL/test, Biolegend, San Diego, CA, USA), anti-GFAP antibody conjugated with APC (0.5 µg/100 µmL, Miltenyi Biotec Inc., Auburn, CA, USA), and anti-S100B antibody conjugated with PE (1 µg/100 µL, LifeSpan Biosciences Inc., Olympia, WA, USA). Cell surface expression of additional SC markers was assessed using anti-NCAM antibody conjugated with PerCP/Cy5.5 (5 µL/test, BioLegend, San Diego, CA, USA), PE-conjugated anti-MAG, and FITC-conjugated anti-MBP antibody (0.4 µg/100 µL, Santa Cruz Biotechnology Inc., Dallas, TX, USA). Antibody dilutions were prepared at the concentrations specified by the suppliers. Flow cytometric acquisition was performed on a Becton Dickinson LSR II instrument (Becton, Dickinson and Company, Franklin Lakes, NJ, USA). Data were analyzed using FlowJo software V9 (FlowJo LLC, Ashland, OR, USA). A minimum of 10,000 events were acquired for each sample, and the experiments were repeated at least three times independently.

### 2.9. Western Blot

SCs (0.25 × 10^6^ cells per 3 mL of complete culture medium with 10% FBS) were incubated in 6-well plates overnight. The culture medium was removed, and the cells were washed with warm, serum-free DMEM. SCs were placed in a 6-well plate with 2.7 mL serum-free DMEM. Then, 0.3 mL serum-free DMEM (control), OCB-free CSF, and OCB-positive SCF samples were added to the culture for 15, 30, and 60 min. To verify the activity of ERK and JNK inhibitors in SCs, cells were first pretreated for 1 h with 10 µM of ERK inhibitor UO126 (Cell Signaling Technology, Boston, MA, USA) or 10 µM JNK inhibitor SP600125 (Santa Cruz Biotechnology Inc.), followed by the addition of SCF samples or control medium for 30 min.

All cells were then collected, washed in PBS, and lysed in 100 μL of RIPA buffer (Alfa Aesar, Ward Hill, MA) containing phosphatase and protease inhibitors for 30 min on ice. After centrifugation (9000× *g*, 15 min, 4 °C), protein concentrations were determined in the supernatants using the Pierce BCA Protein Assay Kit (Thermo Fisher Scientific Inc., Waltham, MA, USA). Ten micrograms of total protein were dissolved in electrophoresis sample buffer, separated on a 4–12% PAGE gel (NuPAGE Bis-Tris, Thermo Fisher Scientific Inc., Waltham, MA, USA), and transferred to a PVDF membrane (Life Technologies, Pittsburgh, PA, USA). After blocking with 5% non-fat milk in TBST, the membrane was incubated with primary antibodies at 4 °C overnight, then with an HRP-conjugated secondary antibody at room temperature. The membrane was processed and treated with the Pierce chemiluminescent reagent (Thermo Fisher Scientific Inc., Waltham, MA, USA). The bands were visualized on a CL-XPosure film (Life Technologies, Pittsburgh, PA, USA) prior to densitometric evaluation of protein levels using UN-SCAN-IT software V7.1 (Silk Scientific, Orem, UT, USA).

The following primary antibodies were used for Western blot assay: Total Erk1/2, c-Jun, Akt (pan), SMAD2, Stat3, and Phospho Erk1/2, c-Jun, Akt, SMAD2, Stat3 (Cell Signaling Technology, Boston, MA, USA). All primary antibodies were diluted 1:1000. Secondary goat anti-mouse and goat anti-rabbit antibodies were diluted 1:100,000 (Invitrogen, Carlsbad, CA, USA). GAPDH expression was evaluated as a housekeeping control using a rabbit monoclonal anti-GAPDH antibody (Cell Signaling Technology, Boston, MA, USA). All Western blot experiments were repeated at least four times.

### 2.10. Statistical Analysis

For the statistical comparison of two groups, Student’s *t*-test was used after assessing normality. If the data were not normally distributed, the Mann-Whitney rank-sum test was performed. To compare multiple groups, a one-way ANOVA was used. SigmaPlot, version 15 (Grafiti USA, Palo Alto, CA, USA) and Prizm, version 10 (Dotmatics, Boston, MA, USA) were used for data analysis and presentation. For all statistical analyses, *p* < 0.05 was considered significant. All experiments were repeated at least twice. Data are presented as the mean ± standard error of the mean (SEM).

## 3. Results

### 3.1. Functional Alterations of Schwann Cells in the MS Microenvironment

**Figure 1:**
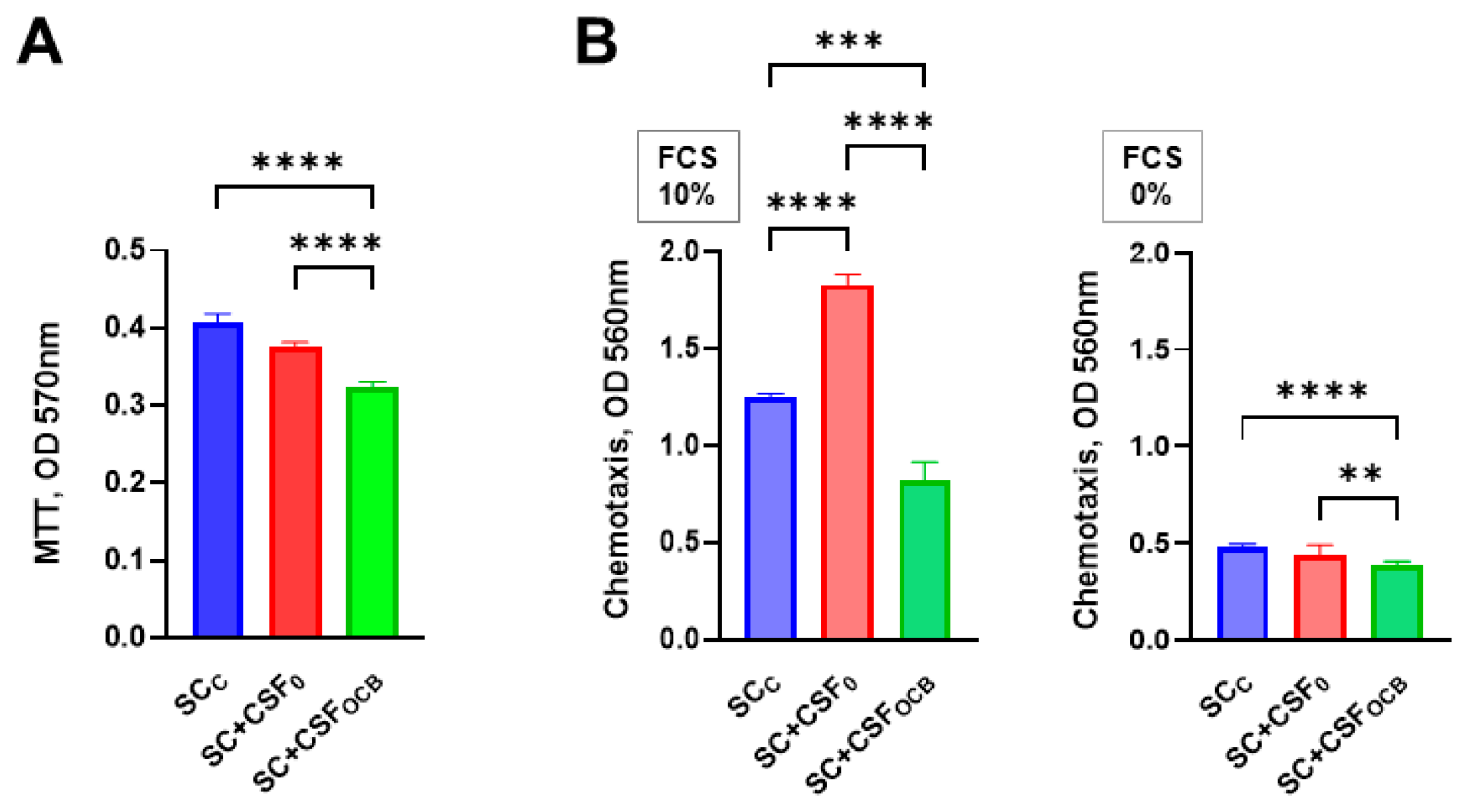
Functional alterations of Schwann cells in the MS microenvironment. Human cultured SCs were treated with 10% (*v*/*v*) CSF from MS patients for 24 h. CSF samples either contain 3+ OCB determined by isoelectric focusing and increased IgG Index (CSF_OCB_) or have no OCB and have a normal (<0.7) IgG Index (CSF_0_). Control SCs were treated with culture medium (SC_C_). SCs were then harvested, and their activity was determined in the MTT assay as described in M&M; results are presented as mean optical density (OD) ± SEM (**A**). The experiment was repeated three times, and a total of 13 CSF samples were tested for each group. Graphics represent all replicates analyzed together. ****, *p* < 0.0001 (one-way ANOVA). The motility of control and CSF-treated SCs was assessed using a colorimetric transwell migration assay, measuring the cells’ ability to penetrate through an 8-µm pore (**B**). SC chemotaxis was determined by their attraction to 10% or 0% FCS in a 24-h assay; results are expressed as OD mean ± SEM ((**B**), **left panel**). Control chemotactic activity of SCs was determined in FCS-free conditions ((**B**), **right panel**). The experiment was repeated 4 times, and a total of 16 CSF samples were tested per group. ****, *p* < 0.0001; ***, *p* < 0.001; **, *p* < 0.01 (one-way ANOVA).

Analysis of the activity of SCs after co-incubation with CSF samples with and without OCB revealed that while OCB-negative CSF does not alter the activity of SCs, OCB-positive CSF significantly decreases SC activation/proliferation in comparison with both control and OCB-negative CSF by up to 15–25% (Figure 1A). Analysis of motility also demonstrated that although control CSF samples up-regulated SC chemotaxis, CSF samples with detectable OCB significantly suppressed SC chemotaxis in comparison with both control and OCB-negative CSF by up to 50–60% (Figure 1B). The chemotaxis of SCs was assessed in medium with and without FCS. Because the results in serum-free medium confirmed inhibition of SC chemotaxis by OCB-positive CSF, although the overall motility of SC was dramatically lower (Figure 1B, right panel), all further experiments were conducted in medium containing FCS. Overall, the analysis of SC function suggests that MS-associated factors present in the CSF of patients with MS may suppress the activity of adult human SCs.

### 3.2. Phenotypic Alterations of Schwann Cells in the MS Microenvironment

**Figure 2:**
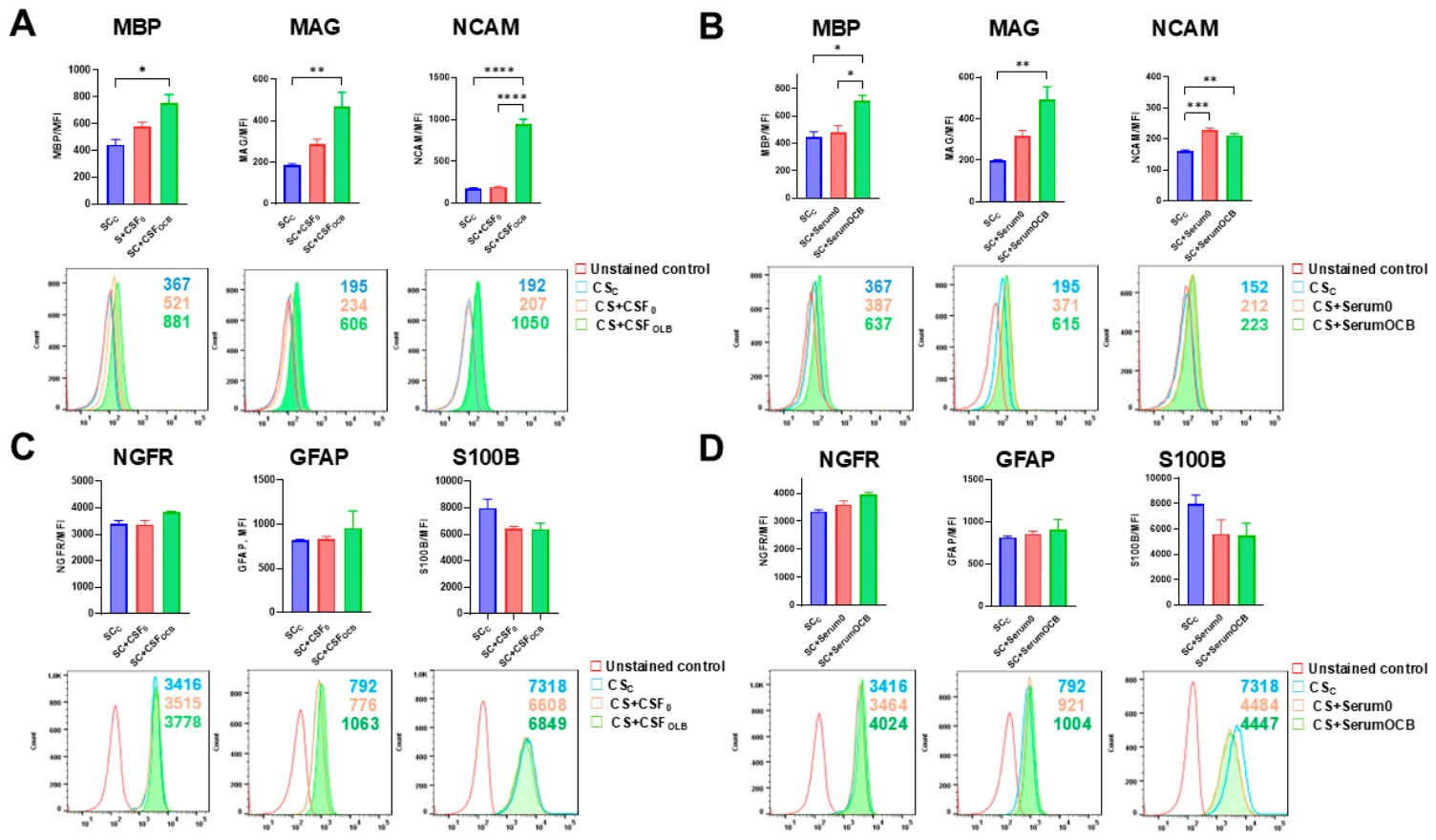
Phenotypic alterations of Schwann cells in the MS microenvironment. Human cultured SCs were treated with 10% (*v*/*v*) CSF or paired serum from MS patients for 48 h. CSF samples either contained 3+ OCB determined by isoelectric focusing and an increased IgG index (CSF_OCB_) or had no OCB and had a normal (<0.7) IgG Index (CSF_0_). Serum samples were from the same patients who had OCB in the CSF (SerumOCB) or had no OCB in the CSF (Serum0). Control SCs were treated with culture medium (SC_C_). SCs were then harvested, and the expression of MBP, MAG, NCAM, NGFR, GFAP, and S100B was determined by flow cytometry as described in M&M. The results of SC treatment with CSF samples are presented as mean fluorescent intensity (MFI) ± SEM from 3 experiments (**A**,**C**) or as original representative results with individual MFI for each group ((**A**,**C**), **lower panels**). A total of 8 CSF samples were tested for each group. ****, *p* < 0.0001; ***, *p* < 0.001; **, *p* < 0.01; *, *p* < 0.05 (one-way ANOVA). The results of SC treatment with serum samples are presented as MFI ± SEM from 3 experiments (**B**,**D**) or as original representative results with individual MFI for each group ((**B**,**D**), **lower panels**). A total of 8 serum samples were tested for each group. Graphics represent all replicates analyzed together.

Analysis of intracellular and surface expression of SC-associated proteins after co-incubation with CSF samples by flow cytometry also revealed that OCB-positive CSF significantly upregulated expression of MBP, MAG, and NCAM in treated SCs (Figure 2A). Mean Fluorescence Intensity (MFI) of NCAM expression on SCs increased more than 5-fold. Positive cell counts confirmed this up-regulation of NCAM expression (Figure 2A, bottom panels). Co-culture of SCs with paired serum samples from patients with OCB-negative and OCB-positive CSF resulted in similar alterations of MBP, MAG, and NCAM expression in SCs, confirming the results of CSF-treated SCs (Figure 2B). No statistically significant changes in the expression of NGFR, GFAP, and S100B were seen in SCs co-incubated with CSF or serum samples (Figure 2C,D). These results confirm that OCB-positive and OCB-negative CSF have differential effects on SCs in vitro.

### 3.3. Gene Expression of Schwann Cell-Associated Regulatory Factors in the MS Microenvironment

**Figure 3:**
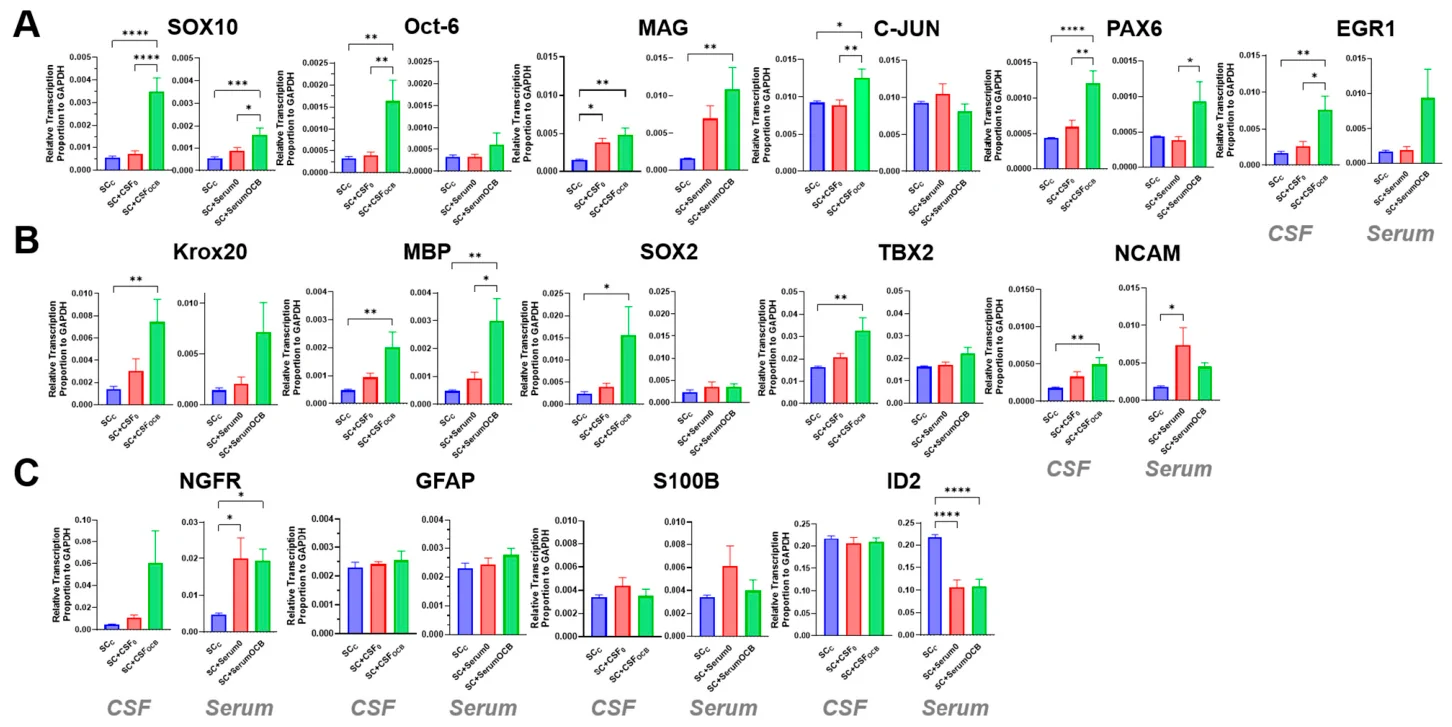
Gene expression of Schwann cell-associated regulatory factors in the MS microenvironment. Human cultured SCs were treated with 10% (*v*/*v*) CSF or paired serum from MS patients for 48 h. CSF samples either contained 3+ OCB determined by isoelectric focusing and an increased IgG index (CSF_OCB_) or had no OCB and had a normal (<0.7) IgG Index (CSF_0_). Serum samples were from the same patients who had OCB in the CSF (SerumOCB) or had no OCB in the CSF (Serum0). Control SCs were treated with culture medium (SC_C_). SCs were then harvested, total RNA was isolated, and qPCR was performed using specific primers as described in the M&M. Results are expressed as relative transcriptional levels, normalized to GAPDH, which was used as a housekeeping control. The mean ± SEM from 4 to 6 independent experiments, using 9–12 CSF and serum samples, is shown. Results are grouped as follows: (**A**) statistically significant differences between the “SC + CSF_OCB_” group and both the “SC + CSF_0_” and “SC_C_” groups; (**B**) statistically significant differences between the “SC + CSF_OCB_” group and the “SC_C_” group; (**C**) no statistically significant differences between the CSF-treated groups. Paired serum sample-treated SC groups are shown in the corresponding linked panels on the right. Graphics represent all replicates analyzed together. ****, *p* < 0.0001; ***, *p* < 0.001; **, *p* < 0.01; *, *p* < 0.05 (one-way ANOVA).

Gene expression of SC-associated proteins and transcription and signaling factors implicated in SC differentiation and repair, or serving as markers of SC activity, was assessed next to verify and expand flow cytometry results. SCs were treated with both CSF and serum samples, and the expression of the genes encoding specific proteins was assessed by qPCR. The results demonstrated that SC co-incubation with OCB-positive CSF resulted in significantly up-regulated expression of SOX10, Oct-6, MAG, c-Jun, PAX6, and EGR1 when compared with SCs treated with the control medium of OCB-negative CSF (Figure 3A). Expression of SOX10, OCT-6, and Pax6 in SCs treated with OCB-positive CSF increased by 7, 5, and 3 times, respectively, in comparison to medium-treated SCs. Expression of Krox20, MBP, Sox2, TBX2, and NCAM in SCs treated with OCB-positive CSF was up-regulated in comparison to medium-treated SCs, but did not reach statistical significance (*p* > 0.05) compared to OCB-negative CSF (Figure 3B). Expression of Krox20 and SOX2 increased by 5.5- and 7-fold, respectively. Treatment of SCs with paired serum samples only partially recapitulated these CSF effects between medium-treated and OCB-positive-treated SCs, suggesting the presence of CSF-specific factors absent in the systemic circulation. Comparison of OCB-negative serum- and OCB-positive serum-treated SCs showed a pattern similar to that in CSF for some factors, with statistical significance only for MBP. Finally, no alteration of the expression of NGFR, GFAP, S100B, and ID2 in SCs treated with either CSF was seen, although the up-regulation of NGFR and down-regulation of ID2 in SCs treated with both types of serum samples were detected. Thus, these results confirmed the flow cytometry findings and suggested that SCs may undergo specific changes within the model MS microenvironment in vitro.

### 3.4. Regulation of Key Signaling Pathways in Schwann Cells in the MS Microenvironment

**Figure 4:**
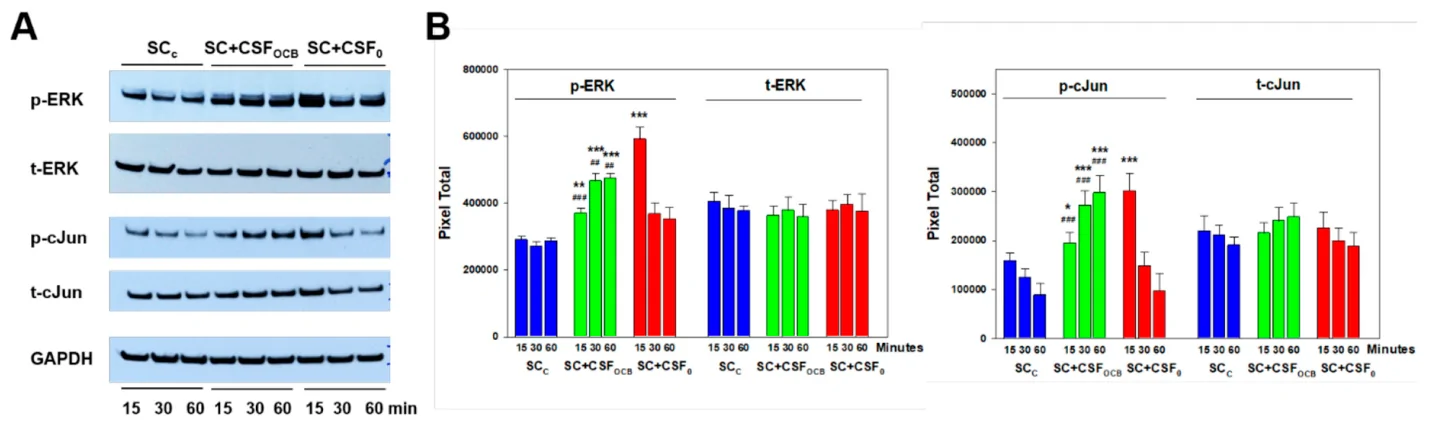
Activation of signal transduction pathways in Schwann cells by CSF. SCs were treated with the control medium (SC_C_), OCB-free CSF samples (SC + CSF_0_), and CSF samples with detectable OCB (SC + CSF_OCB_) for 15, 30, and 60 min. SCs were then harvested, washed in cold buffer, and the expression of the signaling proteins was determined in cell lysates after electrophoretic separation by Western blot, as described in M&M. GAPDH was used as a control. (**A**) results from a representative experiment are shown. (**B**) summary data on the expression of total and phosphorylated ERK and c-Jun in control and CSF-treated SCs from 5 independent experiments, with 14–16 CSF samples per group. All bands were analyzed for protein levels by densitometry using UN-SCAN-IT software, and the results are shown in Total Pixels. ***, *p* < 0.001; **, *p* < 0.01; *, *p* < 0.05 versus corresponding SC_C_ groups (one-way ANOVA). ^###^, *p* < 0.001; ^##^, *p* < 0.01 versus corresponding SC + CSF_0_ groups (one-way ANOVA).

Analysis of Erk1/2, c-Jun, Akt, SMAD2, and Stat3 activation, which are known to regulate SC function during peripheral nerve repair [37], in CSF-treated SC, showed a significant prolongation of ERK1/2 and c-Jun activation in SC treated with OCB-positive CSF compared to both control SCs and OCB-negative CSF-treated SCs (Figure 4). Interestingly, control OCB-negative CSF also induced significant ERK and c-Jun phosphorylation in SCs, but this effect was observed only at 15 min, not at 30 or 60 min. However, OCB-positive CSF upregulated ERK and c-Jun activation at all tested time points, indicating a markedly stronger effect on treated SCs. As expected, there were no differences in the expression of total ERK or c-Jun between the groups. The results also showed no effect of either CSF sample on Akt, SMAD2, and Stat3 activation in SCs, demonstrating a specific effect of CSF on ERK and c-Jun activation in SCs. Thus, these data raised the question of the potential effects of ERK and c-Jun inhibitors on the altered SC function observed in CSF-treated cells.

### 3.5. Prevention of CSF-Induced Schwann Cell Changes by ERK and c-Jun Inhibitors

**Figure 5:**
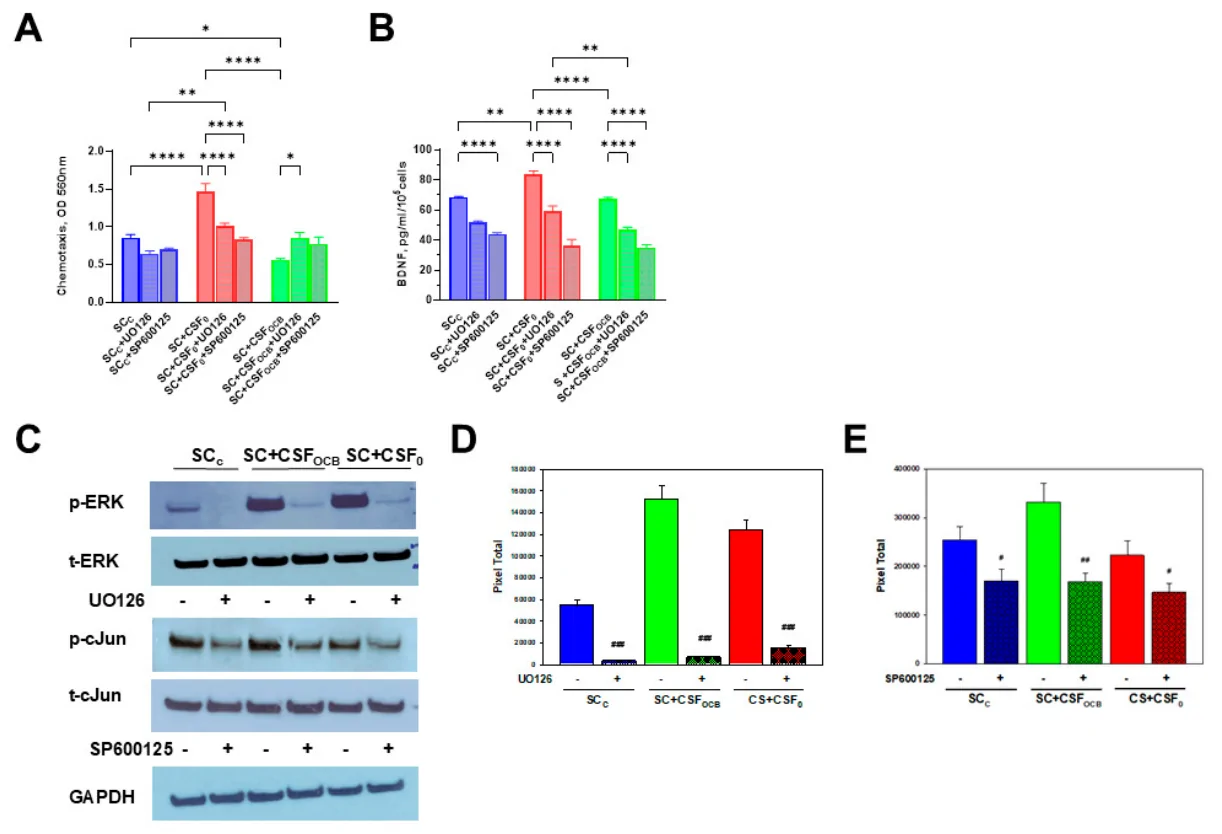
Blockage of CSF-induced changes in Schwann cells. Human cultured SCs were treated with 10% (*v*/*v*) CSF from MS patients for 24 h. CSF samples either contain 3+ OCB determined by isoelectric focusing and increased IgG Index (CSF_OCB_) or have no OCB and have a normal (<0.7) IgG Index (CSF_0_). Cells were pre-treated with 10 mM UO126 and SP600125, inhibitors of the MAPK/ERK and JNK signaling pathways, respectively, for one hour prior to the addition of CSF samples. Control SCs were treated with culture medium (SC_C_). SCs were then harvested, and chemotaxis was determined in a 24-h assay; results are expressed as OD mean ± SEM. Graphics represent all replicates analyzed together. (**A**). The experiment was repeated twice, and a total of 9 CSF samples were tested per group. To assess BDNF production, SCs were pretreated with UO126 and SP600125 and cultured with CSF samples as above. Then, SCs were harvested, washed, and cultured in serum-free medium for 24 h. Cell-free supernatants were collected, and growth factor concentrations were determined by multiplexed assay as described in the M&M. BDNF levels are shown in pg/mL/10^5^ cells as the mean ± SEM. Graphics represent all replicates analyzed together (**B**). The experiment was repeated twice, and 8 CSF samples were tested per group. ****, *p* < 0.0001; **, *p* < 0.01; *, *p* < 0.05 (one-way ANOVA). After pretreatment with UO126 and SP600125 inhibitors, SCs were cultured with CSF samples or control medium for 30 min, then harvested, washed, and lysed to assess the phosphorylation of ERK and c-Jun by Western blot, as described in the M&M. The results of representative experiments assessing ERK and c-Jun expression in control and treated SCs are shown (**C**). The results of two independent experiments, presented as the mean ± SEM of ERK (**D**) and c-Jun (**E**) expression in control and treated SCs, are shown. ^###^, *p* < 0.001; ^##^, *p* < 0.01; ^#^, *p* < 0.05 versus the corresponding “no inhibitor” groups (Student’s *t*-test).

To test the involvement of specific signaling pathways in the effects of CSF on SCs, cells were pre-treated with UO126 and SP600125, MAPK/ERK and c-Jun N-terminal kinase signaling pathway inhibitors, respectively, prior to the addition of CSF samples. Initially, the activity of ERK and JNK inhibitors was assessed by Western blotting to detect their ability to block ERK and c-Jun phosphorylation in CSF-treated SCs (Figure 5). The pretreatment of SCs with UO126 and SP600125 significantly reduced the phosphorylation of ERK and c-Jun, respectively, in cells co-incubated with either CSF samples or control medium (Figure 5C–E). As anticipated, no effect of signaling pathway inhibitors on the expression of total ERK or c-Jun was observed.

As shown in Figure 5A, both the stimulation of SC chemotaxis by OCB-negative CSF and its inhibition by OCB-positive CSF (see Figure 1B) were prevented by both inhibitors or UO126, respectively.

The effect of signaling pathway inhibitors on CSF-treated SCs’ ability to express neurotrophic growth factors was also evaluated. The initial study showed that OCB-negative CSF increased BDNF production in SCs, while OCB-positive CSF had no effect. There were no changes in the production of CNTF, GDNF, or NGF in SCs after incubation with either CSF sample. Figure 5B demonstrates that UO126 and SP600125 effectively blocked the increase in BDNF release from SCs treated with OCB-negative CSF. Although BDNF release in SCs treated with OCB-positive CSF was unaffected, it was significantly reduced by both inhibitors, similar to control SCs, indicating that both medium-treated and CSF-treated SCs were responsive to the inhibition of the MAPK/ERK and JUN signaling pathways.

These proof-of-principle results show that selectively inhibiting specific signal transduction pathways in SCs can prevent CSF-induced changes in their function, including those observed in CSF samples from patients with MS.

## 4. Discussion

The confirmed presence of SCs in the CNS during disease conditions indicates their potential beneficial role in central remyelination [8,38,39]. For example, SC invasion into the spinal cord, brainstem, and cerebellum, along with CNS myelination, has been documented in various animal studies [8]. Postmortem examinations of chronically injured human spinal cords have shown that axonal demyelination can be linked to invading SCs that help restore myelin sheaths around certain spinal axons [9]. Remyelination of CNS axons by SCs has been reported to protect axons, restore axonal conduction, and potentially reverse neurological impairments [40]. These reparative SCs can originate from the PNS or differentiate from adult oligodendrocyte precursor cells [41,42,43,44]. However, SC remyelination of CNS axons may be limited to the entry and exit zones of the spinal root [38], suggesting that SC migration and survival within the CNS might be restricted [45,46]. Additionally, some evidence suggests that SCs within the CNS may exhibit dysplastic phenotypes and reduced longevity [39]. These findings, along with our limited understanding of SC behavior in the pathological CNS environment, pose challenges to the development of SC-based therapies for demyelinating conditions such as MS.

Because increased CSF IgG synthesis relative to serum immunoglobulins (indicated by a higher IgG index and intrathecally produced oligoclonal IgG bands) is considered typical in patients with MS [47,48], we used a model system in which human SCs were incubated in CSF with and without OCB to study SC behavior in the MS microenvironment. We found different effects of OCB-positive and OCB-negative CSF on SC activation/proliferation, migration, neurotrophic factor production, and intracellular signaling, suggesting, for the first time, that these effects should be considered when using SCs for remyelination in MS. Specifically, SC proliferation and chemotaxis were decreased in SCs treated with CSF from MS patients compared to those treated with medium or control (OCB-negative) CSF (Figure 1). SC mobility and migration are important for nerve repair because increased motility enhances SC interactions with other cells in the nerve environment, including other SCs, fibroblasts, and macrophages, influencing SC behavior within the lesion. SC migration also supports axonal regeneration and subsequent myelination of regenerated nerve fibers [49]. Since pharmacologically targeting SCs can improve regeneration after nerve damage [50], we examined inhibitors of the MAPK/ERK and JNK pathways and demonstrated that they blocked CSF-induced changes in SC function (Figure 5).

A component of the transcription factor activator protein-1, c-Jun, is suggested to play a central role in SC reprogramming and plasticity and to serve as a marker of immature, dedifferentiated SCs [1]. It is highly upregulated under pathological conditions such as peripheral nerve injuries, neuropathies, and demyelinating diseases [51]. Jun reduces the myelin transcriptional program, increases the expression of genes that establish the SC repair phenotype, and contributes to SC failure to myelinate axons [2,52]. c-Jun also orchestrates SC support for distressed neurons following mechanical injury and in inherited demyelinating diseases [53]. ERK activation may also lead to demyelination and altered SC proliferation [54]. While our results show c-Jun and ERK1/2 activation in SCs and weakening of SC function in the modeled MS microenvironment, it remains unproven whether SC-induced myelination in the CNS can be suppressed by c-Jun activation. Recent data show that overexpression of JUN in SCs in a mouse model of amyotrophic lateral sclerosis promotes partial demyelination of large- and medium-sized axons, impairs motor performance, and results in a more aggressive disease phenotype [55]. On the other hand, phenotypic and transcriptomic markers alone do not confirm the myelination capability of SCs in different experimental models. Additional studies verifying the sensitivity of SC myelinating properties in the MS environment are needed in functional remyelination assay systems. This should include common in vitro models, such as neurons and SC co-cultures in myelination-promoting media, primary neuron–SC cultures, or organotypic brain slice cultures. Importantly, not only the presence of new myelin but also its biological activity, for instance, improved axonal conduction speed, reduced conduction block, or restored nerve function after remyelination, should also be evaluated.

Decreased activation and motility of SCs treated with MS-CSF may also be explained by up-regulated MAG expression, which is known to be expressed in immortalized SC lines and to inhibit SC motility and proliferation [56]. It is important to note that OCB-positive CSF and paired serum significantly increased MBP expression in SCs (Figure 3). MBP has been reported to induce neurotoxicity through its direct interaction with acidic components on the extracellular surface of the neuronal membrane [57]. These results support our suggestion that SCs should be pharmacologically modified to improve their potential utilization for MS treatment. Furthermore, this also stresses the notion that targeting axo–SC communication may offer additional therapeutic strategies for myelin repair in MS. SC myelination also depends on bidirectional signaling with intact axons and the appropriate formation of the axon-SC unit [58,59]. In degenerating axons in MS lesions, the lack of this cross-talk leaves SCs without the mandatory extrinsic signal required to initiate membrane wrapping [60]. It is impossible, at this moment, to discern the impact that SC-specific signaling defects in the MS environment may have on axons, which is itself demanding on SC as it would be necessary to block neuronal access to SC signaling without preventing normal SC remyelination.

We demonstrated that JNK and ERK inhibitors may prevent MS-related inhibition of SC chemotaxis and BDNF production. Overexpression of c-Jun in SCs may be linked to increased GDNF levels, a marker specific to repair SCs [61]. However, we did not observe an increase in GDNF expression in CSF-treated SCs. Conversely, OCB-free CSF enhanced BDNF secretion by SCs, unlike OCB-positive CSF (Figure 5), indicating that SC function might be suppressed in the MS microenvironment. SC-derived BDNF is crucial for axon growth, regeneration, and remyelination [62,63,64,65]. BDNF levels increase in proliferating, migrating SCs and during myelination, and can promote myelination in SCs [66,67,68]. These findings align with our results, which show that MS-associated CSF factors inhibit BDNF up-regulation in SCs compared with control CSF. However, these in vitro findings require further testing in the in vivo MS models.

Importantly, although it is clear that SC-derived trophic factors are key molecules that promote axonal regeneration after PNS nerve injury, there is no consistent evidence in the literature that links the different neurotrophic profiles of human SCs to specificity for both PNS and CNS axon regeneration. Denervated SCs secrete a plethora of neurotrophic factors that are key to promoting axonal growth and regeneration. This includes BDNF, NGF, GDNF, PTN, HGF, and TGF-β, among others [69]. For instance, BDNF has been shown to increase CNS myelination by directly activating oligodendrocytes [70]. Since the type of nerve injury, and thus SC injury, determines the profile of secreted neurotrophic factors by SCs, future studies should focus on identifying factors that show changes in the experimental models of the MS microenvironment.

Furthermore, while c-Jun is an important transcription factor that regulates the repair SC identity [71], the zinc-finger transcription factor Krox20 is a key regulator of myelinating SCs [72]. A cross-inhibitory association between Krox20 and c-Jun has been established, and high levels of c-Jun hinder Krox20 amplification in SCs, while high levels of Krox20 lower the phosphorylation of c-Jun by JNK [73,74]. However, in our study, elevated expression of c-Jun and Krox20 was observed in OCB-positive CSF-treated SCs (Figure 3). This suggests that in the MS environment, SCs may lose their dedifferentiated or maturation-associated phenotypic patterns, which could be characterized as an SC injury response or as malformed adaptive cellular reprogramming [5]. Other results of our study support this hypothesis.

For example, the expression of pro-myelinating genes, including *Krox-20*, *Oct-6*, *Sox10*, and *MBP* [1], was increased in SCs co-incubated with OCB-positive CSF samples in parallel with up-regulated expression of negative regulators of myelination, such as *c-Jun*, *Sox2*, and *Egr-1* (Figure 3). Thus, the classic pattern of regulators of SC myelination polarity is not observed in SCs treated with OCB-positive CSF. *SOX10* is expressed throughout the entire SC lineage [75] and is a defining marker of repair SCs. Pax6 is detected in early SC development, in immature and proliferating SCs [76,77]. Oct6 is indicative of the promyelinating stage. *Sox2* is expressed in SC precursors and immature SCs [78]. *Sox2* expression declines as SCs undergo differentiation [79], and the re-initiation of *Sox2* expression in repair SCs reduces myelination and increases proliferation [80].

Expression of the immature SC marker NGFR was not affected by CSF treatment of SCs. Similarly, we did not observe altered GFAP expression in treated SCs, a factor associated with SC reprogramming to a dedifferentiated state, and also observed in SCs in axonal neuropathies [81]. Transcription factor Id2, known to negatively regulate myelin differentiation [82], was also not changed in CSF-treated SCs (Figure 3).

While MS-treated SCs display markers of SCs observed in nerve injury, they do not exhibit the expected signaling profile or ‘classic’ repair phenotype. At the same time, MS-treated SCs exhibit unusual activation of a number of transcription factors and suppressed functional activity. Interestingly, gene expression analysis of the SC lineage showed that dedifferentiated SCs in the injured nerve lacked a transcription factor profile characteristic of a specific stage in the SC lineage [83]. These data also support our conclusion that SCs undergo abnormal adaptive cellular reprogramming in the MS microenvironment.

In summary, we found that human SCs undergo significant functional, phenotypic, and signaling changes in an in vitro model of the MS microenvironment. As SC function may be markedly down-regulated and critical signaling pathways are up-regulated, analysis of detected abnormalities and SC markers indicates that SCs do not exhibit either a ‘classic’ dedifferentiation-repair-like phenotype or a complete maturation phenotype when placed in MS-like conditions. They demonstrate an uncommon pattern of cellular signaling reprogramming, associated with decreased motility and potentially decreased myelination. Importantly, this SC injury response can be partially prevented by inhibitors of c-Jun and ERK1/2. However, the effect of the MS environment on SC myelination ability remains to be determined. Validation of our first proof-of-principle results using primary SCs would be important to exclude cell line-specific effects. The CSF components responsible for the observed SC phenotype must be identified, and a direct assessment of SC remyelination or myelinating capacity in the model MS environment is required. These studies are currently in progress in the laboratory.

Further studies using different EAE models are required to verify the potential therapeutic role of modified SC-based therapy in CNS remyelination. Importantly, these EAE studies should include several models induced by active immunization with encephalitogenic peptides or proteins or by the administration of CNS antigen-specific lymphocytes, as well as both CD4^+^ and CD8^+^ T cell–dependent EAE models [84]. These studies should determine whether pharmacologically modified SCs can serve as a new therapeutic approach for treating CNS demyelinating diseases.

## 5. Conclusions

A growing body of evidence from preclinical EAE models and clinical studies has shown that natural or inducible remyelination can repair damaged CNS regions in MS, and that both resident and transplanted SCs in demyelinated areas significantly enhance myelin repair and regeneration [20,21,22,23,27]. Together with the development of optimized SC generation protocols and the promising successes of SC transplantation now in clinical trials, these data support the suggestion that SCs and SC-like cells are among the most promising cell subsets for cell-based therapies [2,28,85,86].

Although decades of research have demonstrated the therapeutic value of SCs in various PNS and CNS diseases in rodents, non-human primates, and humans [5,87], SC implantation requires an understanding of SC behavior and longevity within the abnormal CNS microenvironment induced by MS progression. To date, no data have described potential functional alterations and corresponding molecular mechanisms in adult nerve-derived SCs placed in the MS microenvironment. Therefore, our study aimed to provide the first proof of principle that MS-associated factors in CSF and serum can alter the crucial function of human SCs by regulating specific signal transduction pathways. By analyzing the phenotypic, functional, and molecular characteristics of human SCs in the model MS microenvironment, we revealed that SC proliferation and motility were suppressed, while the expression of both pro-myelinating genes and negative regulators of myelination was up-regulated. This unusual pattern of cellular signaling reprogramming of the SC injury response was associated with active phosphorylation of ERK and c-Jun and could be partially prevented by inhibitors of c-Jun and ERK1/2.

These results provide the first evidence that therapeutic SCs may lose functionality in the MS environment and suggest that they should be protected or modified before delivery to the diseased or injured brain. We also demonstrated the opportunity to identify the signal transduction pathways that may hinder or permit SCs to take control of central axon remyelination and provide persistent SC integration, survival, and prolonged remyelination. Our model of the MS microenvironment, together with our results, provides an avenue for identifying CNS factors that regulate SC longevity and motility in the central parenchyma, such as myelin components. This is crucial for designing reasonable treatment approaches for the use of SCs in CNS repair to complement oligodendrocyte remyelination and reduce axonal loss.

Our data suggest that SC transplantation strategies for treating MS and other demyelinating pathologies of the CNS may be limited by the regenerative capacity of SCs in a damaged CNS environment, providing a rationale for the development of clinical trials using autologous PNS-derived SCs. Thus, a systematic understanding of SC behavior in MS-associated pathophysiological conditions should provide a practical tool for controlling and directing the regenerative process and lay the groundwork for developing new glia-centric treatment approaches to counteract axonal loss, pain, and neuroinflammation.

## Data Availability

The original contributions presented in this study are included in the article. Further inquiries can be directed to the corresponding author.
